# Increasing serum miR-223-3p indicates the onset, severe development, and adverse prognosis of bronchiectasis: a retrospective study

**DOI:** 10.1186/s12890-024-03170-y

**Published:** 2024-07-22

**Authors:** Jia Fang, Yao Xu, Chenghui Lin, Jiewen Yang, Dongxu Zhai, Qingyuan Zhuang, Wangli Qiu, Yun Wang, Longjuan Zhang

**Affiliations:** 1 Respiratory Medicine Center, The First Dongguan Affiliated Hospital of Guangdong Medical University, No. 42, Jiaoping Road, Tangxia Town, Dongguan, 523710 China; 2 Medical Laboratory Center, The First Dongguan Affiliated Hospital of Guangdong Medical University, Dongguan, 523710 China; 3 Department of Emergency, The First Dongguan Affiliated Hospital of Guangdong Medical University, Dongguan, 523710 China; 4 Department of Gastroenterology, The First Dongguan Affiliated Hospital of Guangdong Medical University, Dongguan, 523710 China; 5 Department of Clinical Pharmacy, The First Dongguan Affiliated Hospital of Guangdong Medical University, Dongguan, 523710 China; 6 Department of Ultrasonography, The First Dongguan Affiliated Hospital of Guangdong Medical University, No. 42, Jiaoping Road, Tangxia Town, Dongguan, 523710 China

**Keywords:** Diagnosis, Prognosis, Inflammation, Severity, Trachea and bronchial

## Abstract

**Background:**

miR-223-3p has been demonstrated as a *Pseudomonas aeruginosa* colonization-related miRNA in bronchiectasis (BE), but its clinical value in BE has not been revealed, which is of great significance for the clinical diagnosis and monitoring of BE. This study aimed to identify a reliable biomarker for screening BE and predicting patients’ outcomes.

**Methods:**

The serum expression of miR-223-3p was compared between healthy individuals (*n* = 101) and BE patients (*n* = 133) and evaluated its potential in distinguishing BE patients. The severity of BE patients was estimated by BSI and FACED score, and the correlation of miR-223-3p with inflammation and severity of BE patients was evaluated by Pearson correlation analysis. BE patients were followed up for 3 years, and the predictive value of miR-223-3p in prognosis was assessed by logistic regression analysis.

**Results:**

Significant upregulation of miR-223-3p was observed in BE patients, which significantly distinguished BE patients and showed positive correlations with C-reactive protein (CRP), procalcitonin (PCT), interleukin 6 (IL-6), and neutrophil-to-lymphocyte ratio (NLR) of BE patients. Additionally, miR-223-3p was also positively correlated with BSI and FACED scores, indicating its correlation with inflammation and severity of BE. BE patients with adverse prognoses showed a higher serum miR-223-3p level, which was identified as an adverse prognostic factor and discriminated patients with different prognoses.

**Conclusion:**

Increasing serum miR-223-3p can be considered a biomarker for the onset, severity, and prognosis of BE.

## Background

Bronchiectasis (BE) is a kind of pathological injury in the trachea and bronchial tissues. The basic clinical symptoms are recurrent cough, phlegm, and even hemoptysis [1]. Dilatations of the airways is the main feature of BE, which is pathological injury and is irreversible, continuous, and permanent with a long-term course of disease, which has a serious influence on patients’ daily lives [2]. The onset of BE involves various factors, including the chronic colonization of bacteria and airway inflammation [3]. The clinical diagnosis of BE is mainly based on radiological examinations, and the severity was also defined based on the radiological findings. However, due to the complex disease conditions and the similar symptoms with other trachea and bronchial diseases, the radiological diagnosis of BE is always incomplete and lacks prediction of disease development and outcomes [4]. On the other hand, the employment of radiological examination is limited to patients’ conditions and is not suitable for all patients. The early detection of BE is of great clinical importance, and exploring easily detected and reliable biomarkers for BE would help predict patients’ prognosis and provide a reference for the early intervention and adjustment of therapeutic efficiency, which has become a research hot point and challenging problem in the clinic [5].

microRNAs (miRNAs) are a series of small non-coding RNAs produced by the processing of single-stranded RNA precursor by the Dicer enzymes, which have been demonstrated to play vital roles in various life activities. Several studies have also reported the functional role of miRNAs in the occurrence of BE and revealed their regulatory mechanisms underlying disease progression. *Pseudomonas aeruginosa* is one of the most important pathogenic bacteria, of which the colonization would induce severe development and result in adverse prognosis of patients [6, 7]. A previous study identified several miRNA candidates correlated with *P. aeruginosa* colonization in the sputum of BE, where miR-223-3p was included [8]. miR-223-3p has been suggested to mediate the disease course of various respiratory diseases, including asthma, acute respiratory distress, chronic obstructive pulmonary disease, and COVID-19-induced pneumonia [9–12]. Additionally, miR-223-3p was also revealed to regulate inflammation in human diseases, which seriously affected the severity of BE [13–15]. Therefore, according to previous evidence, miR-223-3p was hypothesized of great potential to diagnose BE and predict patients’ outcomes, which lacks confirming data.

This study enrolled a group of BE patients with healthy individuals as the control group. Through comparing serum miR-223-3p levels and summarizing the severity and prognosis data of BE patients, the significance of miR-223-3p in discriminating BE and predicting patients’ prognosis was disclosed, aiming to identify a novel biomarker for BE.

## Methods

### Enrollment of study subjects

A total of 133 BE patients were enrolled from The First Dongguan Affiliated Hospital of Guangdong Medical University from January 2018 to December 2020. The diagnosis of BE was based on criteria for adults with the help of high-resolution computed tomography (HRCT) examinations [16], combined with the following clinical symptoms: (1) cough, sputum, and dyspnea; (2) chest pain, different degrees of hemoptysis, loss of appetite, fever, anemia, weight loss, and fatigue; (3) lung moist rales can be heard, and some patients were with thick dry rales or clubbing toes. The signs for diagnosing BE by HRCT included direct signs and indirect signs. The direct signs were: (1) the inner diameter of bronchus/accompanying pulmonary artery over 1 cm; (2) the bronchus did not taper from the center to the periphery; (3) a bronchial shadow observed 1 cm from the peripheral pleural or close to the mediastinal pleura. The indirect signs were: (1) thickening bronchial wall; (2) mucous impaction; (3) “mosaic” sign or “gas trapping” observed by expiratory CT; (4) columnar or sac-like changes, tracheowall thichening (the bronchial inner diameter < 80% of outer diameter) and tree bud signs. Enrolled patients should be clearly aware and cooperative and had never received treatments of anti-infection, bronchodilator, or leukotriene inhibitors within 3 months prior to the study. Patients combined with malignant tumors, lung diseases, immune deficiency, or severe hemoptysis in the lungs were excluded.

Another 101 healthy individuals were enrolled in the physical examination center of our hospital during the same period. All participants had signed informed consent, and the study had obtained approval from the Ethics Committee of The First Dongguan Affiliated Hospital of Guangdong Medical University. BE patients were followed up for 3 years to summarize the outcomes of patients. Adverse complications, such as pulmonary heart disease, respiratory failure, and emphysema, and deaths were defined as adverse outcomes.

### Sample collection and preparation

Fasting elbow venous blood was collected from all study subjects. A part of the blood samples was centrifugated at 4000 g for 15 min to isolate serum, and another part was stored in an anti-coagulation tube used for the analysis of inflammation indicators, including, C-reactive protein (CRP), procalcitonin (PCT), interleukin 6 (IL-6), and neutrophil-to-lymphocyte ratio (NLR).

### Analyses of inflammation indicators

The levels of CRP (C-Reactive protein ELISA Kit, ab260058, Abcam, USA), PCT (Procalcitonin ELISA Kit, ab221828, Abcam, USA), and IL-6 (IL-6 ELISA Kit, ab178013, Abcam, USA) were analyzed by enzyme-linked immunosorbent assay performed on whole blood samples with corresponding kits according to the manufacturers’ instructions.

NLR was calculated based on the counts of neutrophil and lymphocyte measured by a Sysmex XN-10 Automated Hematology Analyzer (Sysmex GmbH, Germany).*Evaluation of BE severity*.

The severity of BE was evaluated by the bronchiectasis severity index (BSI) and FACED scores according to previous studies [17–19]. BSI score summarized the features of patients, including age, BMI, the time of acute exacerbations within 1 year, the time of hospitalization within 2 years, the percentage forced expiratory volume in one second (FEV1) of the estimated value, the number of lobe segments involved in imaging, modified medical research council dyspnea scale, the colonization of *P. aeruginosa* and other bacteria. The total score is 26 points, where 0–4 points indicate mild BE, 5–8 points indicate moderate BE, and over 9 points indicate severe BE.

The FACED score included similar features with a total score of 7 points, where 0–2 points indicate mild BE, 3–4 points indicate moderate BE, and 5–7 points indicate severe BE.

### Real-time quantitative PCR

Total RNA was extracted from serum using QIAzol Lysis Reagent (QIAGEN, Germany) and evaluated by the ratio of OD260/280 (ranging from 1.8 to 2.2). Reversed transcription was conducted with a Reverse Transcription kit (QIAGEN, Germany) to generate cDNA. Then, amplification was performed on the ABI 7900 PCR system (Applied Biosystem, USA) with SYBR Green. The relative expression of miR-223-3p was calculated with the 2^−ΔΔCT^ method with cel-miR-39 as the internal reference.

### Statistical analyses

All data have been confirmed in accordance with the normal distribution by a P-P plot using SPSS 23.0 software. A difference comparison between the two groups was performed with a student’s t-test (*P* < 0.05). The receiver operating curve (ROC) was performed to evaluate the significance of miR-223-3p in distinguishing BE patients and discriminating patients with different prognoses, and the risk factors for adverse prognosis of BE patients were identified by logistic regression analysis. The diagnostic cutoff, sensitivity, and specificity of miR-223-3p was obtained at the maximum Yuden index of ROC. The correlation of miR-223-3p with inflammation and severity of BE patients was assessed by Pearson correlation analysis.

## Results

### Upregulated miR-223-3p distinguished BE patients from healthy individuals and indicated disease severity

**Fig. 1:**
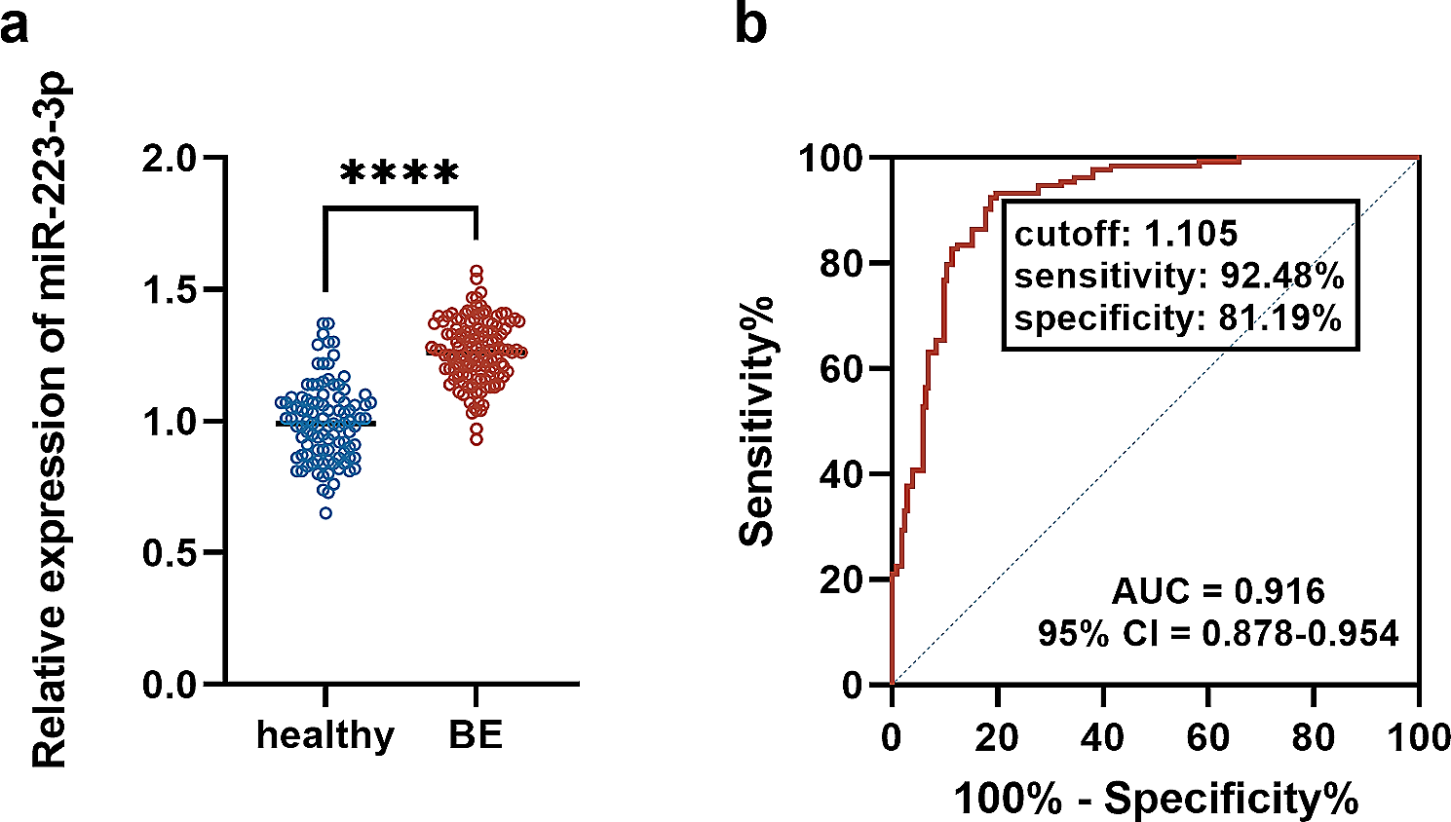
Expression and diagnostic value of miR-223-3p in BE. (**a**) serum expression. (**b**) ROC curve. ^****^P < 0.0001

Healthy individuals included 66 males and 35 females with an average age of 59.59 ± 7.77 years, while BE patients were composed of 88 male patients and 45 female patients with an average age of 59.80 ± 9.06 years. The age and gender composition are not statistically different (data not shown). Significant upregulation of miR-223-3p was observed in BE patients relative to healthy individuals (Fig. 1a), which discriminated BE patients with a relatively high sensitivity (92.84%) and specificity (81.19%) indicating by ROC with the cutoff of 1.105 (AUC = 0.916, Fig. 1b).

**Fig. 2:**
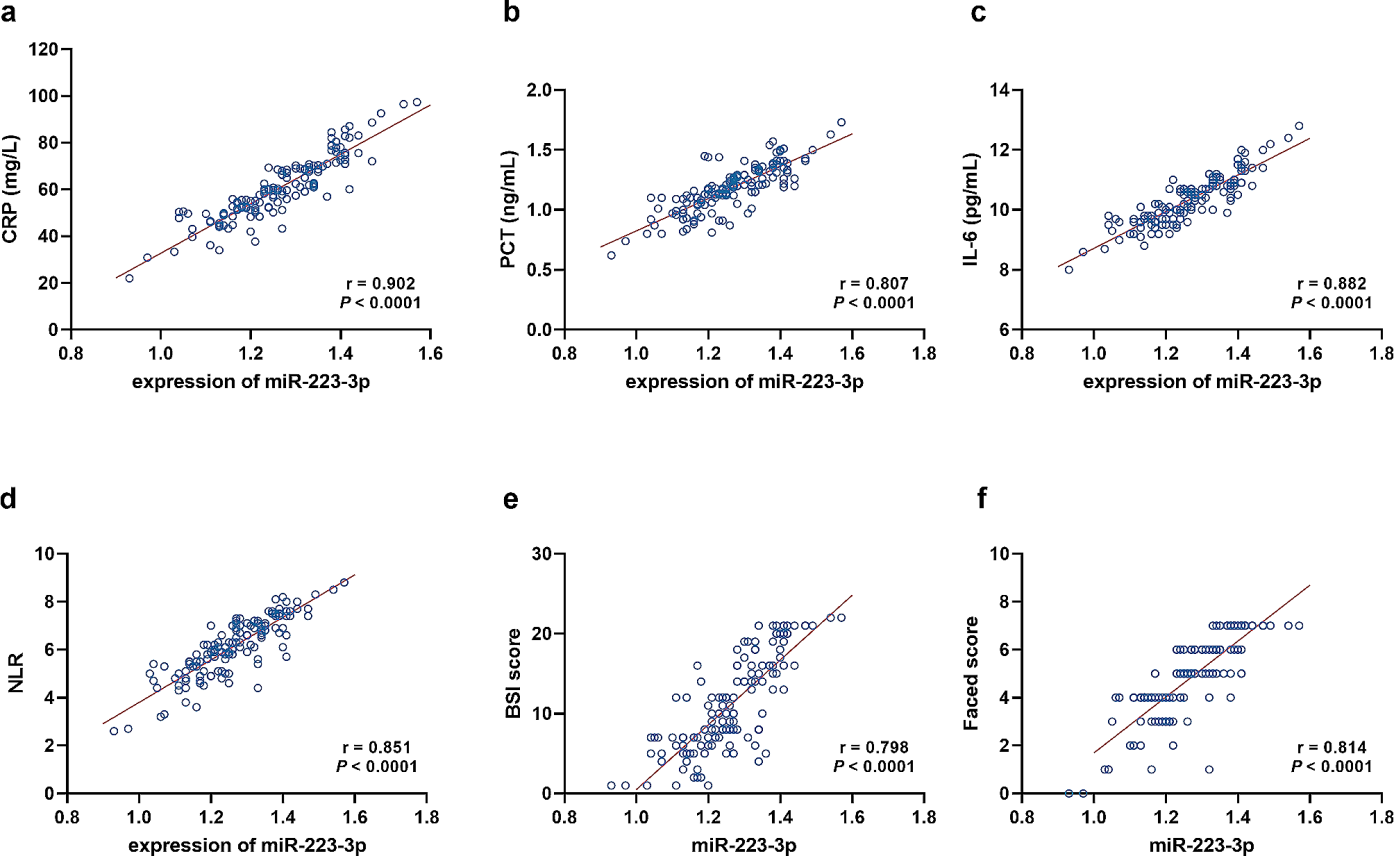
Correlation of miR-223-3p with inflammation and severity. a-d. correlation with inflammation indicators, CRP (**a**), PCT (**b**), IL-6 (**c**), NLR (**d**). **e**-**f**. correlation with severity indicators, BSI score (**e**), and FACED score (**f**)

The inflammation of BE patients was evaluated by the levels of CRP, PCT, IL-6, and NLR. Significantly positive correlations of miR-233-3p with these indicators were observed with the correlation coefficients of r_CRP_ = 0.902 (Fig. 2a), r_PCT_ = 0.807 (Fig. 2b), r_IL−6_ = 0.882 (Fig. 2c), and r_NLR_ = 0.851 (Fig. 2d). While positive correlations were also revealed in the severity of BE with the serum miR-223-3p levels. miR-223-3p showed positive correlation with the BSI score (*r* = 0.798, Fig. 2e) and FACED score (*r* = 0.814, Fig. 2f).

### Increased serum miR-223-3p levels predicted the adverse prognosis of BE patients

**Fig. 3:**
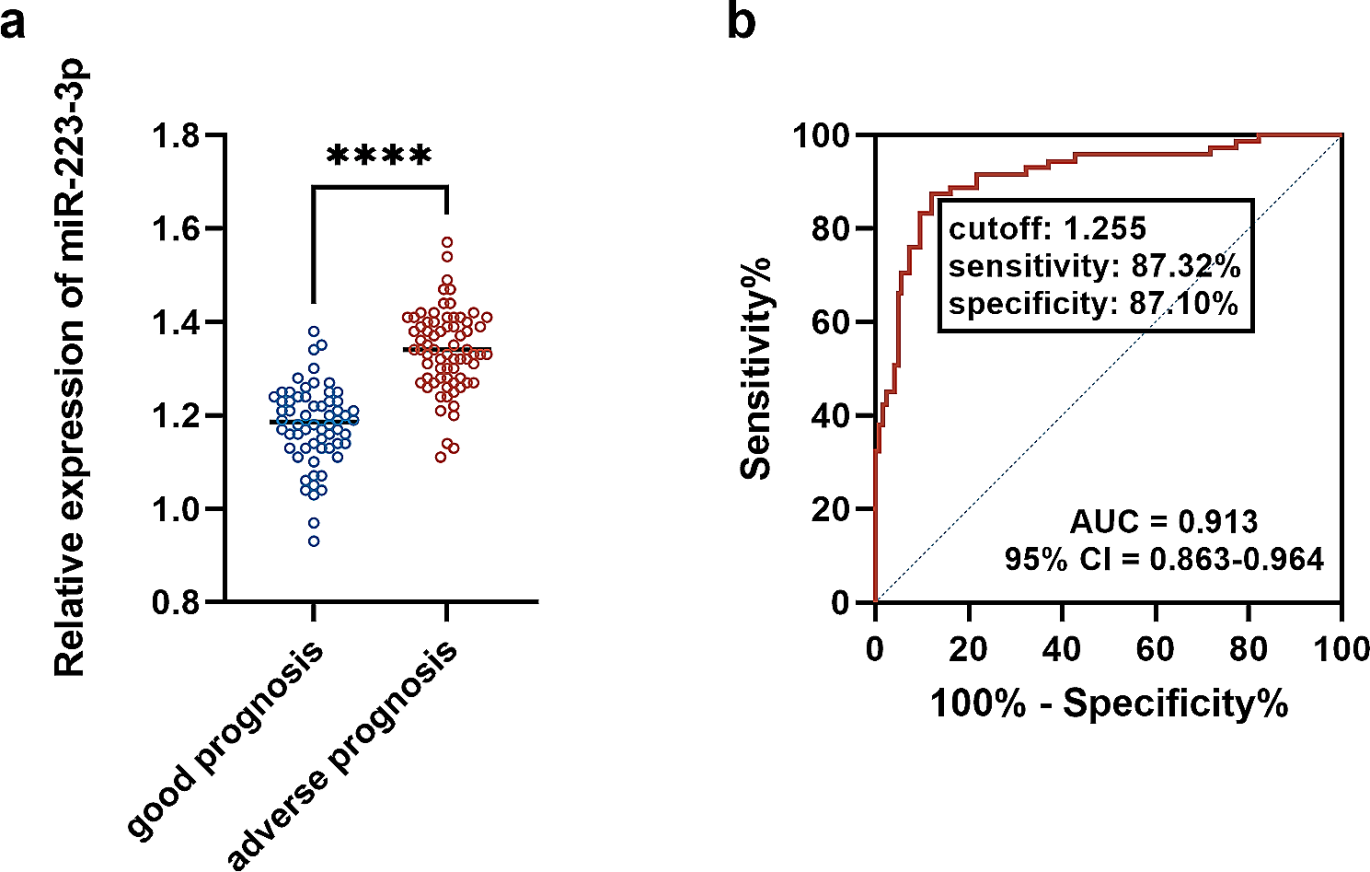
Expression and correlation of miR-223-3p with prognosis of BE patients. (**a**) serum expression. (**b**) ROC curve. ^****^P < 0.0001

According to the 3-year follow-up data, patients were divided into a good-prognosis group and an adverse-prognosis prognosis group. Patients with adverse prognoses showed a higher PCT level (*P* = 0.021), BSI score (*P* = 0.008), and FACED score (*P* = 0.012) than that of patients with good prognoses. CRP, IL-6, and NLR showed no significant difference between the two groups (Table 1). The serum miR-223-3p was relatively higher in patients with adverse prognoses than patients with good prognoses (Fig. 3a). Serum miR-223-3p also showed significant diagnostic value in discriminating patients with adverse prognoses with the cutoff of 1.255 (Fig. 3b). Logistic regression analysis demonstrated that miR-223-3p was the most significant risk factor for the adverse prognosis of BE patients (OR = 16.224, *P* < 0.0001), as well as BSI score (OR = 2.919, *P* = 0.28), FACED score (OR = 2.751, *P* = 0.035), PCT (OR = 2.395, *P* = 0.048), and NLR (OR = 2.551, *P* = 0.049, Table 2).

**Table 1:** Baseline information of BE patients with different outcomes

	Good-prognosis	Adverse-prognosis	*P*-value
Age	59.26 ± 9.41	60.27 ± 8.77	0.523
Gender (male/female)	40/22	48/23	0.707
BMI	23.51 ± 3.89	24.70 ± 4.26	0.098
CRP	58.93 ± 7.69	62.16 ± 17.37	0.179
PCT	1.14 ± 0.17	1.22 ± 0.21	0.021
IL-6	10.24 ± 0.69	10.39 ± 0.92	0.291
NLR	5.94 ± 0.98	6.32 ± 1.39	0.068
BSI score			0.008
0–4	10	3	
5–8	19	16	
> 9	33	42	
FACED score			0.012
0–2	7	4	
3–4	20	23	
5–7	35	44	

**Table 2:** Logistic regression analysis evaluating risk factor for adverse prognosis in BE patients

	OR	95% CI	*P*-value
Age	1.542	0.648–3.670	0.328
Gender	0.643	0.263–1.572	0.333
BMI	1.329	0.569–3.103	0.511
BSI score	2.919	1.123–7.590	0.028
FACED score	2.751	1.076–7.034	0.035
miR-223-3p	16.224	5.618–46.852	0.000
CRP	1.599	0.649–3.936	0.307
PCT	2.395	1.007–5.696	0.048
IL-6	1.707	0.640–4.550	0.285
NLR	2.551	1.005–6.471	0.049

OR: odd ratio; BMI: body mass index, kg/m^2^; BSI: bronchiectasis severity index; CRP: C-reactive protein, mg/L; PCT: procalcitonin, ng/mL; IL-6: interleukin 6, pg/mL; NLR: neutrophil-to-lymphocyte ratio

## Discussion

### Significance of miR-223-3p in diagnosing BE patients

Exploring accurate, convenient, efficient, and non-invasive diagnostic biomarkers for BE has always been an urgent problem to solve in the clinic. miR-223-3p is a key regulator in respiratory diseases that screens onset, predicts patients’ survival, regulates disease development, and modulates inflammation [20–24]. Blood analysis is a common non-invasive examination for primarily screening human diseases. Here, increasing serum miR-223-3p level was observed in BE patients and showed significant value in distinguishing BE patients. The obtained diagnostic cutoff for miR-223-3p in BE was 1.105. It means patients with a serum miR-223-3p over 1.105 are considered of high property of BE. However, there were some healthy individuals showed higher serum miR-223-3p over the cutoff in the study subjects of the present study. The AUC value of ROC (0.916) indicates the diagnostic efficiency, therefore, serum miR-223-3p cannot totally diagnose BE patients. Additionally, the relatively small sample size is not large enough to confirm miR-223-3p as a diagnostic index including in clinical practice, the observed increasing level of miR-223-3p might assist in improving the accuracy and specificity of screening BE, which can be verified in the clinic.

### Correlation of miR-223-3p with inflammation in BE patients

Inflammation indicators have been considered effective means to screen infectious diseases and evaluate the disease conditions [25]. CRP, PCT, IL-6, and NLR are sensitive inflammation indicators. According to clinical experiences, CRP, PCT, and IL-6 showed rapid increase in the early stage of infection [26, 27]. The increase of PCT is earlier and is specific to bacterial infection, fungal infection, sepsis, and multiple organ failure [28, 29]. The sensitivity of IL-6 is higher than CRP and PCT [30]. NLR directly represents the balance between neutrophils, lymphocytes, and inflammatory activators and could further indicate infection or acute inflammation. Moreover, NLR has been demonstrated as a biomarker for BE [31]. There have been studies that combined these inflammation indicators and found combining could improve the diagnosis of pulmonary infection and help predict disease severity. A previous study demonstrated increasing miR-223-3p in leukocytes was positively correlated with IL-17, which is critically involved in inflammation and mediates immune response [9]. The inflammatory indicators were positively correlated with the serum miR-223-3p levels in BE patients, suggesting its ability to assess the degree of inflammation and potential to predict disease severity.

### Correlation of miR-223-3p with the severity of BE patients

The study employed two assessing scales for the severity of BE patients. Although the features and indicators are similar between the BSI score and the FACED score, the clinical significance of the two scores is slightly different [32]. BSI score shows more potential in predicting deterioration, hospitalization, health status, and death, while FACED score emphasizes predicting future acute exacerbations and risk of hospitalization [33]. Therefore, BSI showed a higher significance in predicting the adverse prognosis of BE patients than the FACED score in the present study. A positive correlation of miR-223-3p was observed with both two scores, indicating miR-223-3p is of great significance in predicting severe development and adverse outcomes of BE patients, which was further validated based on the follow-up data. However, this study only focused on the correlation of miR-223-3p with patients’ disease severity, overlooking the changes in miR-223-3p when the severity progressed. Therefore, there is a need for a dynamic study on the expression of miR-223-3p during the progression of BE, which would provide more reference for the clinical application of miR-223-3p in monitoring BE.

### Correlation of miR-223-3p with the prognosis of BE patients

With the 3-year follow-up survey, over half of BE patients experienced adverse outcomes, including pulmonary heart disease, respiratory failure, emphysema, and even death. Pulmonary heart disease is one of the most common complications of BE, due to lung hyperinflation, increased hypoxemia, and systemic inflammation during the onset of BE. The pathological injuries in the trachea and bronchial tissues dramatically influenced the work of the respiratory system, and therefore, respiratory diseases are also frequent complications in BE [34]. Although this study had not differentiated different complications in patients with adverse prognoses, the significance of miR-223-3p was demonstrated. The serum miR-223-3p levels were higher in patients with adverse prognosis and were identified as a risk factor for adverse prognosis. As expected, the increasing serum miR-223-3p level discriminated patients with good prognosis and adverse prognosis sensitively and specifically. Therefore, besides predicting severe disease development, serum miR-223-3p could also be considered an indicator of the unfavorable outcomes of BE. The significance of miR-223-3p in specific complications needs further investigations to completely reveal the prognostic value in BE.

Serum miR-223-3p showed great significance in screening and prognosis prediction of BE, and it can be considered an auxiliary indicator for suspected patients or patients not available for radiological examination. Compared with protein analyses, the quantification of serum miR-223-3p by PCR is quicker and more convenient, but the diagnostic cutoff needs further larger-sample-size investigations.

## Conclusions

In conclusion, the upregulation of serum miR-223-3p could indicate the onset of BE, severe disease development, and adverse prognosis, suggesting the potential of serum miR-223-3p in diagnosing and prognosis predicting of BE.

## Data Availability

The datasets used and/or analysed during the current study are available from the corresponding author on reasonable request.

## Abbreviations

BE: Bronchiectasis
BSI: Bronchiectasis severity index
CRP: C-reactive protein
FEV1: Forced expiratory volume in one second
HRCT: High-resolution computed tomography
IL-6: Interleukin 6
miRNAs: MicroRNAs
NLR: Neutrophil-to-lymphocyte ratio
PCT: Procalcitonin
ROC: Receiver operating curve
